# Multiple loss-of-function variants of taste receptors in modern humans

**DOI:** 10.1038/srep12349

**Published:** 2015-08-26

**Authors:** K. Fujikura

**Affiliations:** 1 Kobe University School of Medicine, 7-5-1, Kusunoki-cho, Chuo-ku, Kobe 650-0017, Japan

## Abstract

Despite recent advances in the knowledge of interindividual taste differences, the underlying genetic backgrounds have remained to be fully elucidated. Much of the taste variation among different mammalian species can be explained by pseudogenization of taste receptors. Here I investigated whether the most recent disruptions of taste receptor genes segregate with their intact forms in modern humans by analyzing 14 ethnically diverse populations. The results revealed an unprecedented prevalence of 25 segregating loss-of-function (LoF) taste receptor variants, identifying one of the most pronounced cases of functional population diversity in the human genome. LoF variant frequency in taste receptors (2.10%) was considerably higher than the overall LoF frequency in human genome (0.16%). In particular, molecular evolutionary rates of candidate sour (14.7%) and bitter (1.8%) receptors were far higher in humans than those of sweet (0.02%), salty (0.05%), and umami (0.17%) receptors compared with other carnivorous mammals, although not all of the taste receptors were identified. Many LoF variants are population-specific, some of which arose even after population differentiation, not before divergence of the modern and archaic human. I conclude that modern humans might have been losing some sour and bitter receptor genes because of high-frequency LoF variants.

Taste is a primitive sense that helps recognize and distinguish key dietary components^1^,^2^,^3^. Humans perceive these tastants through sensory organs called taste buds, primarily located on the tongue with a few on the palate and throat^1^. The sensation of taste is classified into five prototypical categories: sweet, bitter, sour, salty, and umami^1^,^2^,^3^,^4^,^5^,^6^. In addition recent compelling evidence raises the possibility of an additional sixth and seventh taste modality devoted to the perception of calcium^7^,^8^ and lipids^9^,^10^. Taste perception often affect nutrient intake and appetite, and thus taste decrements can lead to food poisoning and over-exposure to hazardous chemicals^1^,^11^.

During the last 10 years, the discovery and characterization of mammalian candidate taste receptors has progressed tremendously^2^,^3^,^4^,^5^,^6^. Recent studies have provided an intriguing example that extensive losses of receptor genes is widespread among many species and that taste receptors directly shape feeding behavior^12^,^13^,^14^. Thus the taste receptors are likely to be a highly plastic gene family showing frequent gene birth/loss events during mammalian evolution.

There are some evidences that variation in taste perception within the human population is correlated with polymorphisms of taste receptors^15^,^16^,^17^,^18^,^19^,^20^,^21^. For example, the *hT2R43* W35 allele makes people most sensitive to the bitterness of the natural plant toxins, aloin and aristolochic acid, and an artificial sweetener, saccharin, compared with S35 allele^17^. In addition, a closely related gene’s *hT2R44* W35 allele is also sensitive to saccharin^17^. Individuals who do not possess these allele do not taste the bitterness of these compounds at low micromolar concentrations^17^. Another well-known example is *hTAS2R38*^18^. African populations have higher levels of genetic, geographic and phenotypic diversity at the *TAS2R38* locus^18^. *TAS2R38* P49A, A262V and V296I have clear effects on the phenylthiocarbamide (PTC) sensitivity in humans^18^. Polymorphisms of hTas1R taste receptor genes are also important for taste variation^20^,^21^. These polymorphisms illustrate the influence of recent genetic variation on a common trait. Yet the whole spectrum of interindividual differences of taste sensitivity has remained to be fully elucidated.

One clear signature of gene evolution is loss-of-function (LoF) variants caused by nonsense mutations. The pioneering study by Wang *et al.* identified 80 human-specific nonprocessed pseudogenes^22^. Later on, Yngvadottir *et al.* genotyped 805 LoF variants and found that more than 10 of them appears to be subject to adaptive selection^23^. Recently MacArthur *et al.* applied filters to 2951 putative LoF variants obtained from 185 human genomes and validated 565 out of 1111 LoF variants^24^. The 1000 Genomes (1000G) project also revealed that SNVs can lead to differences in functional and non-functional genes between modern humans, including instances of rescue from pseudogene to functional gene^25^. In this research, I made a large-scale attempt to investigate the recently arose LoF mutations of taste receptors in modern humans using two population exome sequences, and analyzed whether the most recent of these disruptions may still segregate with the intact forms. The results here revealed an unprecedented prevalence of segregating LoF variants in taste receptors, one of the most pronounced cases of functional population diversity in the human genome.

## Results

### Profiles of human taste receptor gene variations reveal striking individuality

The representative taste receptors are composed of more than 50 coding regions distributed in clusters over most chromosomes in mammals^4^. In mice, taste receptor pseudogenes comprise 15% of this gene range; in humans, a fraction roughly two times larger appears to be inactivated^4^. Of more than 50 taste receptor genes, more than half appear to have been pseudogenized by mutations during mammalian evolution^4^. Extreme diminution of the functional taste receptor repertoire was a relatively recent genomic process and is probably still ongoing^4^. Therefore, I conjectured that a substantial fraction of modern human taste receptors may segregate between intact and pseudogene forms.

**Figure 1:**
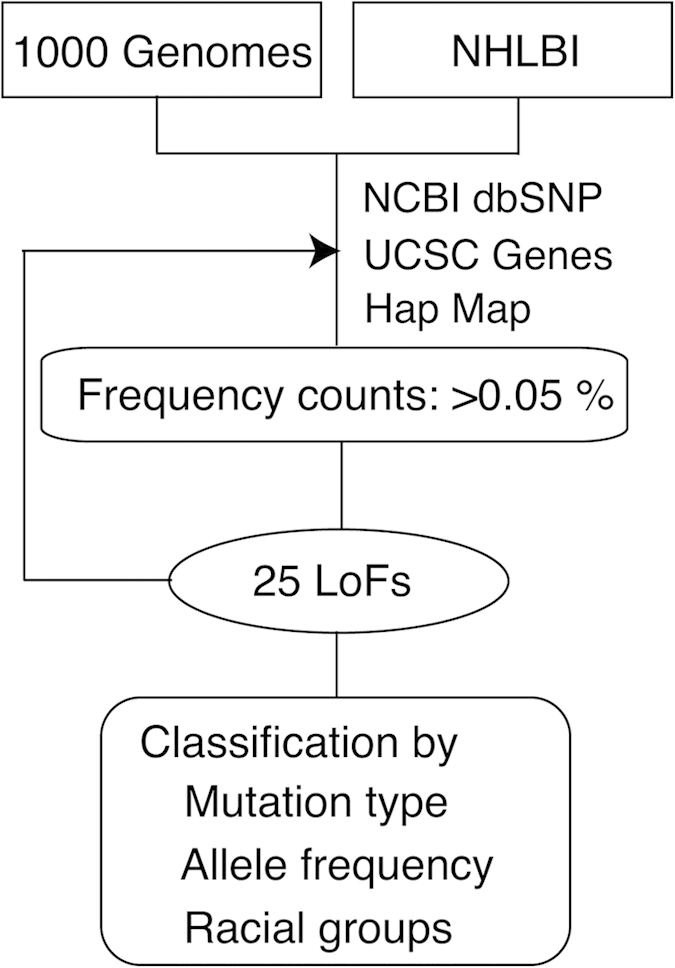
Strategy for identification of deleterious mutations within taste receptor genes. A flow chart used to identify the sequence variations that have harmful influence on human taste senses shows 25 LoF variants within taste receptor genes. Several platforms (NCBI dbSNP, UCSC Genes, and Ensemble variation) were used to access the validity of variants and examine previous gene annotations.

**Figure 2:**
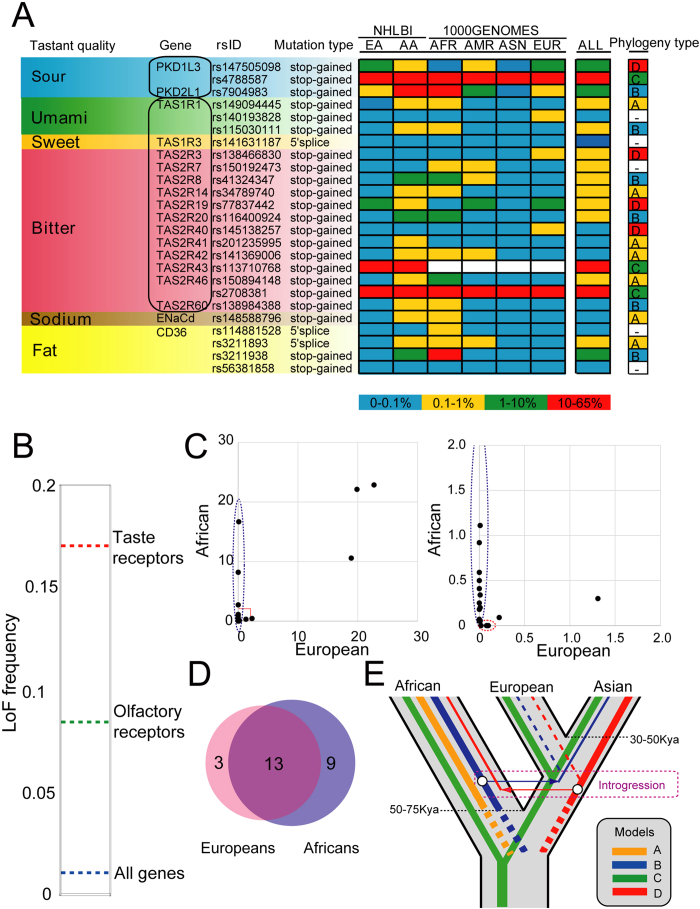
Loss-of-function variants of taste receptors in major ethnic groups. (**A**) Allele frequency of LoF variants of 18 taste genes in European (EA) and African (AA) American (NHLBI), and African (AFR), Ad mixed American (AMR), East Asian (ASN) and European (EUR) (1000G). Colored boxes indicate the frequency of LoF variants (blue, 0–0.1%; yellow, 0.1–1%; green, 1–10%; red, 10–65%).Taste genes are ordered according to their tastant quality and functions. dbSNP ID and disruption type is described. (**B**) Taste receptors are frequently altered by nonsense mutations. Blue, Green and red lines indicate average LoF frequency of taste receptors (2.10%), olfactory receptors (1.41%) and overall genes of human genome (~0.16%), respectively. (**C**) Comparison of MAF (%) distribution of taste receptor genes between African and European. Vertical and horizontal axis indicate MAF in African and European, respectively. Right panel is enlarged view of the red box at the left panel. Blue and red dotted circle indicate the population-specific or enriched variants. (**D**) Venn diagram showing the number of shared and unique LoF variants for taste receptor genes between European American and African American. (**E**) Hypothetical origins of different taste between ethnic groups. Divergence of taster and nontaster alleles before and after race divergence is indicated as four lines (phylogeny type A, B, C, D).

I focused on three kinds of LoF variants expected to correlate with complete loss of function of the affected transcripts: one stop codon–introducing (nonsense) or two (5′ and 3′) splice site disrupting single-nucleotide substitutions. 1000G^25^ and NHLBI databases^26^ were searched for variations with the potential to affect the protein integrity of taste receptor genes (Fig. 1 and Table S1). Target receptor genes are as follows: *TAS1R1*, *TAS1R2*, *TAS1R3*, *TAS2R1*, *TAS2R3*, *TAS2R4*, *TAS2R5*, *TAS2R7*, *TAS2R8*, *TAS2R9*, *TAS2R10*, *TAS2R13*, *TAS2R14*, *TAS2R16*, *TAS2R19*, *TAS2R20*, *TAS2R30*, *TAS2R31*, *TAS2R38*, *TAS2R39*, *TAS2R40*, *TAS2R41*, *TAS2R42*, *TAS2R43*, *TAS2R45*, *TAS2R46*, *TAS2R50*, *TAS2R60*, *PKD1L3*, *PKD2L1*, *CD36*, *ENaCa*, *ENaCd*, *HCN1*, and *HCN4* (Table S1; Note that these genes are positioned as major candidate taste receptors in this research). Taste receptor are different from olfactory receptors (ORs) in that taste receptors are not composed of single gene family^2^,^3^,^4^. The data matrix included 7,595 individuals from six ethnic origins (Table S2). I searched for LoF variants in taste receptor from these datasets and extrapolated that the number of segregating LoF variants in the entire human genome was 25, of which 18 were expected to have a Minor allele frequency (MAF) of >0.05% (Fig. 2A). The total frequencies of LoF alleles divided by the number of taste receptor genes (2.10%; TAS2R^18^ = 1.76%; PKD-like (PKD1L3 + PKD2L1^2^,^3^ = 14.7%) was extremely higher than the overall average LoF frequency in the human genome (approximately 0.16% (calculated from LoF variants caused by nucleotide substitution; this proportion also includes both LoF frequency of taste and smell receptor genes)) (Fig. 2B). Except for complete pseudogenes, LoF frequency of taste receptor genes exceeded those of olfactory receptor (OR) genes (approximately 1.41%; recalculated from 367 functional ORs because more than half genes analyzed in a past study^27^ have already been proved to be complete pseudogenes; If the mutation frequency of pseudogenes is calculated, their MAF is regarded as 100% and mutation frequency become quite high).

In theory, a combination of these genes could give rise to an enormous series of individual differences in taste perception; each examined individual had a unique genotypic pattern. The average number of LoF sites in hTAS1R, hTAS2R and PKD-like (*PKD1L3* + *PKD2L1*) receptors per individual was 0.00371, 0.422 and 0.294, respectively. Coupled with TAS and PKD-like genes, *CD36* (fat receptor^9^,^10^), and *ENaCd* (*SCNN1D*) (sodium channel^28^,^29^,^30^,^31^) were frequently lost in some populations (Fig. 2A, Table S2 and S3). In contrast, I confirmed that the other taste buds-specific or -enriched genes previously reported^32^,^33^ had been rarely lost (45 genes analyzed are described in Supplementary note 1).

Using 1000G sequences, I confirmed that the three pseudogenized bitter receptors *TAS2R2P*, *TAS2R62P*, and *TAS2R64P*^34^ have never been functionally restored by gain-of-function mutations in any human population. No novel taste receptor genes were acquired after the divergence of chimpanzees and humans. These surveys of LoF variants in taste receptor genes pointed out the possibility that modern humans might have continued to genetically lose the repertoire of receptor genes after species differentiation.

### Population-specific LoF variants in taste receptor genes

Because of physical barriers to migration, ethnic populations rarely interbreed as convenient in theoretical random models. Consequently populations from different ethnic groups often have different genetic backgrounds and therefore different frequencies of genetic polymorphisms^35^. To examine the genetic distance of taste receptor genes among populations, I investigated whether frequencies and distributions of mutations had regional and racial biases by constructing a map for relationships between LoF frequency and gene locations (Figure S1 and Table S2 and S3). Comparative analyses showed that patterns of nucleotide substitution rates varied substantially among different regions of the genome and among different ethnic populations (Figure S1 and Table S2 and S3).

These results suggested that some taste receptor variants had different origins and emphasized the necessity to narrow the range of target sequences that are to be searched on the basis of the ethnic background. Of note, African samples provided a huge resource for discovering variant sites, whereas non-African individuals had significantly fewer variations (Fig. 2C,D and Table S2). Furthermore, the non-African diversity was largely a subset of the African diversity (Fig. 2C,D and Table S2).

By comparing alleles among individuals from various ethnic backgrounds, I further estimated the extent of differentiation in taste receptor genes before and after the divergence from African origins^36^ approximately 50,000–75,000 years ago. A pair-wise proportions test^37^ was performed, which is used for testing a null hypothesis stating that proportions in two populations are the identical. This is referred to as a z-test because the statistic is


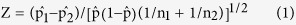


where pˆ = (p_1_ + p_2_)/(n_1_ + n_2_), and the indices^1^,^2^ refer to the first and second line of the Table.

The LoF events of taste receptors into four phylogenies (types A, B, C, and D) was divided based on a significant difference (*P* < 0.05) between populations, suggesting that humans might have always been losing their taste receptors, even after population differentiation (Fig. 2A,E). Moreover, these results revealed that gene mutation flow from Africans to non-Africans, and vice versa, had occurred (Fig. 2E). This evidence suggested that the general theory^19^,^38^,^39^ that different evolutionary pressures, such as diets, toxins, and climates (energy consumption) have shaped the different chemosensory repertoires in mammals might be applicable to modern human populations.

**Figure 3:**
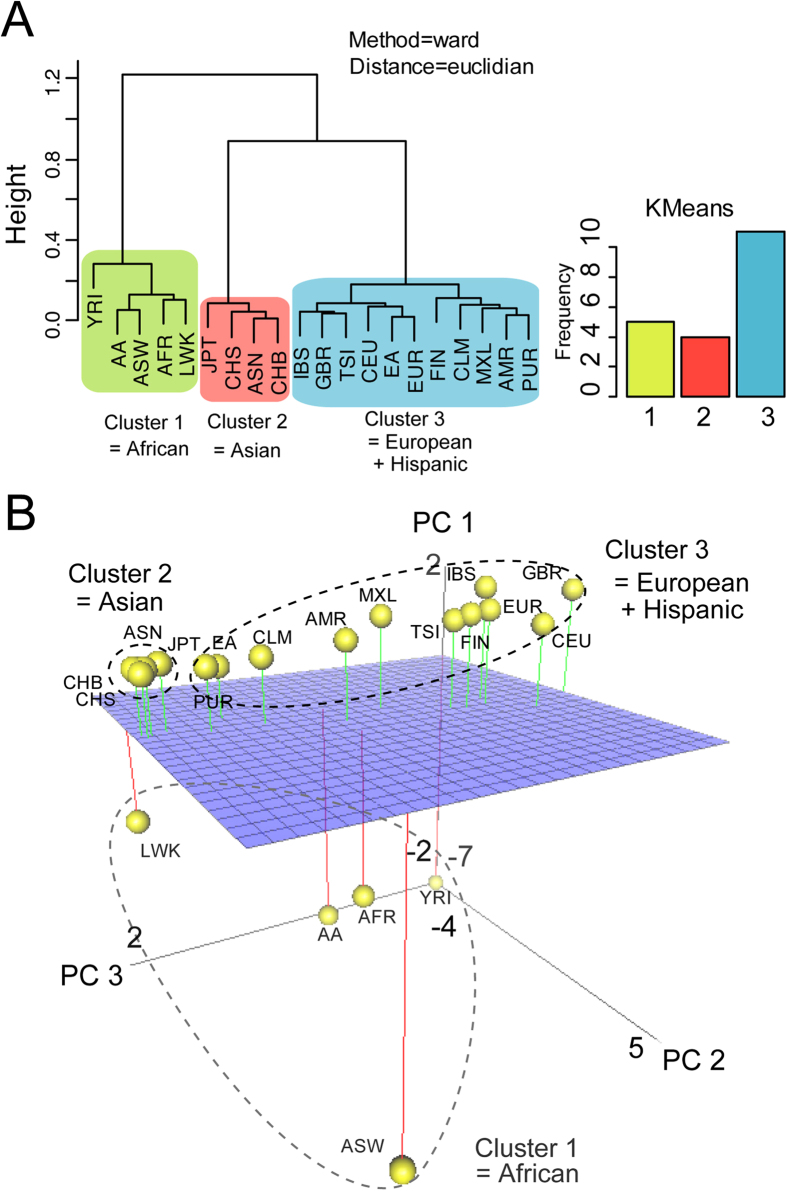
Multivariate analysis of CYP genetic variants across 14 + 6 ethnic populations. (**A**) Hierarchical (left dendrogram; ward’s algorithm) and Non-Hierarchical (right bar graph; k-means algorithm) clustering of taste receptor variants produce three genetic clusters across 14 + 6 ethnic populations. African subpopulations: Yoruba in Ibadan, Nigeria (YRI), Luhya in Webuye, Kenia (LWK) and African ancestry in Southwest USA (ASW). East Asian subpopulations: Han Chinese in Beijing, China (CHB), Southern Han Chinese (CHS) and Japanese in Tokyo, Japan (JPT). Hispanic subpopulations: Colombians from Medellin, Colombia (CLM), Puerto Ricans from Puerto Rico (PUR) and Mexican Ancestry from Los Angeles, USA (MXL). European subpopulations: Iberian population in Spain (IBS), Toscans in Italy (TSI), British in England and Scotland (GBR), Finnish in Finland (FIN) and Utah residents with Northern and Western European ancestry (CEU). (**B**) PCA projection of samples taken from a set of 14 + 6 ethnic groups. Grey-scaled spheres correspond to Asian, African, European and Hispanic.

I next investigated population-by-population differences in taste LoF variants. To separate confused data sets to make several distinct classes, multivariate analysis was used for the matrices of spectra from 14 ethnic groups to compare their profiles of taste receptor gene variations (Fig. 3A, left panel; see also Figure S2). Hierarchical Ward’s method showed that 14 ethnic populations could be categorized into three general groups: African, Asian, and European–Hispanic. Hierarchical median algorithms (Fig. 3A and S3) and non-Hierarchical clustering algorithms (k-means algorithms; Fig. 3A, right panel) also supported these categories.

Furthermore, principal component analysis (PCA) was used to compare the LoF variants of taste receptor genes among various countries (Fig. 3B). The three principal components (PC 1, 2 and 3) reflected the difference of taste LoF variants in these populations. These structure patterns were consistent between the two approaches (Fig. 3A,B). However, when using the population-by-population approach, there were large amounts of differences in taste LoF variants among African, European and Hispanic populations (Fig. 3B). In contrast, taste genetic structures differed only slightly among Asian populations (Fig. 3B). The taste LoF patterns in Hispanics was genetically closer to those in Europeans than the other populations, and some Hispanic (PUR and CLM) and African (LWK) groups are similar to Asian populations (Fig. 3B). East Asian groups, including Japanese and Chinese, had only a few LoF variants of bitter receptor genes (Table S3). These results illustrated population-by-population genomic signatures for the taste LoF variants in humans and sufficient proof that taste receptor genes had evolved further in individual colonized areas.

### Independent origins of mutant alleles

Previous studies raise the possibility that different ethnic groups often shared same taste sensitivity (for example, sweet, salt, bitter and so on) to various compounds mediated by taste receptors^40^,^41^,^42^,^43^. Yet, phylogenetic relationships among LoF taste receptor variants were, for the most part, consistent with the hypothesis that some LoF alleles had independently arisen at least twice between the two ethnic groups over the course of human evolution (Fig. 2A). For example, both rs150894148 and rs2708381 caused stop-gain mutations in *TAS2R46* at almost the same position, although their origins were speculated to be different (rs150894148: phylogeny type A; rs2708381: phylogeny type C; Fig. 2A).

**Figure 4:**
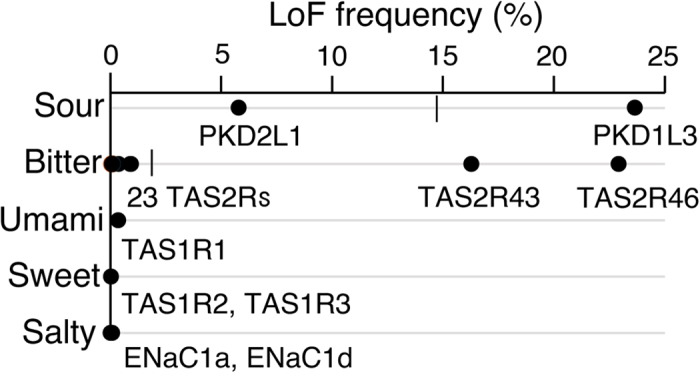
Molecular evolutionary rate of five taste senses in modern humans. LoF mutation frequency of each taste receptor is shown in circle. Average LoF frequency of taste receptor genes of two senses (sour and bitter) are shown in vertical lines.

The molecular evolutionary rates of sour and bitter receptor genes seems much higher than those of the other receptor genes in any ethnic group (Fig. 4). In general sour or bitter tastes can be unpleasant, but—because many toxic substances taste sour or bitter—they can also be life-saving. The result here raised the possibility that modern humans lacking sour and/or bitter taste receptors would not seemingly be at a significant disadvantage. At present not all of the taste related genes were analyzed, and thus confirmation will have to wait until further study is conducted. Characterizing these patterns could facilitate us to unveil the evolutionary pressure acting on modern humans.

### Archaic origin of LoF variants in taste receptor genes

To further address the origin of taste variants, I explored how modern humans acquired the highly polymorphic taste receptor genes in comparison with the archaic humans Neanderthals^44^ and Denisovans^45^, a likely sister groups to the modern human. The same sets of taste receptor genes were present in both Neanderthals and Denisovans, suggesting that they were not subject to any strong multiallelic balancing selection. Virtual genotyping of Denisovan and Neanderthal genomes showed no sign of pseudogenization and thus great similarities with current humans (Table S4M). Intriguingly, even the most frequent mutation rs123321 was not present in Denisovan and Neanderthal genome (Table S4).

I quantitatively estimated the allele age of each mutation. On the assumption of a constant population, the range of allele age was from 0.2 ± 3.3 kiloyears (±s.d.) to 96.3±92.7 kiloyears (Table S4). Recent studies using demographic model^46^ also provided the allele age in the similar order of magnitude (Table S4). At present the divergence time of Denisovans, Neanderthals and modern humans from their most recent common ancestor is calculated to be 640,000 years^47^. Therefore I concluded that most LoF variants in taste receptors in modern human were likely to be acquired after the divergence of the modern and archaic humans.

## Discussion

In this study the widespread occurrence of a recent decline in the number of functional taste receptors (pseudogenization) was first illustrated. This report might raise the hidden possibility that individual taste differences may be driven, in part, by LoF variants in taste receptors. Genetic taste variation was first reported in 1931 as differences in the ability of humans to taste PTC^48^. Although a pile of information about the genetics of taste thresholds has been accumulated for more than 80 years^15^,^16^,^17^,^18^,^19^,^20^,^21^,^49^,^50^, but the whole spectrum of interindividual differences of taste sensitivity still remain unknown. Of course, LoF variants in taste receptors not necessarily mean taste impairment because I can consider some scenarios of widespread pseudogenization. For instance, recent study suggested that many hTAS2R receptors share a similar chemical ligands and conversely one agonist can activate several hTAS2Rs, illustrating that hTAS2Rs cooperatively play a common role in toxin avoidance^51^,^52^. Some specific taste receptors may have been lost in the human as a result of their functional redundancy. An alternative possibility is that there might be currently no more serious threat to toxins and chemical compounds.

Massive loss of taste receptors that I reported is rather unusual, and only a few analogous cases have been described. Another pronounced case is olfaction, which has also undergone a recent decline in the number of functional genes after population differentiation^27^. Olfactory receptors are scattered across the human genome and many genes have already been pseudogenized by nucleotide substitutions^27^. Olfaction and taste receptors cooperatively contribute to flavor, and thus are speculated to have progressed and regressed together during vertebrate evolution^53^,^54^. They may have evolved by affecting each other with high expansion rates in addition to selective pressures due to partial functional redundancy^27^,^53^,^54^. The high-frequency genetic polymorphisms are unique to taste and olfactory receptors in the human genome. This co-evolution could comprise a hitherto unexplored aspect of human genotypic heterogeneity and could be a major landmark of human evolution.

Recent reports provided some examples that non-synonymous mutations cause interindividual differences of taste sensitivity^15^,^38^,^55^,^56^,^57^. For instance, non-synonymous polymorphisms in *TAS1R1* and *TAS1R3* cause variations of sensitivity to L-glutamate (Umami)^15^,^55^. In addition Africans have higher levels of genetic, geographic and phenotypic diversity at the *TAS2R16* and *TAS2R38* locus^38^,^56^,^57^. These reports demonstrated that non-synonymous mutations also largely altered bitter and umami taste sensitivity among same populations.

Copy number variants (CNVs) have been reported for *TAS2R43* and the ***−**45* locus^58^. CNVs could result in both overrepresentation and absence of expressed proteins, and have the potential to exert extreme effects on phenotypes. At present, firm conclusions cannot be drawn and more research on genotype–phenotype associations should provide insights into the function of each taste receptor.

The results here demonstrated that the LoF variant frequency in taste genes, especially sour- and bitter-related receptors, is likely to be extremely high (Figs 2, 4 and Table S2) although not all of the taste receptors were uncovered. Sour and bitter tastes are characteristics of many toxic compounds^59^ and have a survival advantage^19^. The result here raised the possibility that modern humans lacking sour and/or bitter taste receptors would not seemingly be at a significant disadvantage. In ancient times, meals that may contain toxic substances were life-threatening for human ancestors^59^, but modern humans may have no need to worry about the risks of daily exposures to toxicants, leading to the high-frequency LoF variants in taste receptor genes. Recent studies have also provided an intriguing example that a loss of sweet receptor genes is widespread among carnivorous species and that taste receptors directly shape feeding behavior^12^,^13^,^14^. In contrast to carnivorous mammals, the frequent loss of sweet receptor genes was not observed in human lineage.

Relationships among the taste perception, polymorphisms, and dietary choices still remain unclear. Further genetic, biochemical and cell biological studies are necessary to uncover the total effect of mutations on individual taste sensitivity; they should lead us to a fundamental understanding of the modern human’s taste.

## Materials and Methods

### Analysis of LoF variants from diverse ethnics

Sequence data was collected and analyzed from 1000G (http://browser.1000genomes.org/index.html) and NHLBI (http://www.nhlbi.nih.gov/) database. Briefly, these data-sets consisted of high-coverage whole-genome and exome sequence data from diverse ethnic groups, respectively. In addition NCBI dbSNP (http://www.nlm.nih.gov/), UCSC genome browser (http://genome.ucsc.edu/) and HapMap (http://hapmap.ncbi.nlm.nih.gov/) was used as sequencing platform, which were analyzed using an integrated read mapping and variant-calling pipeline to generate initial catalogue of candidate LoF variants of taste receptor genes. Each MAF (%) was calculated using excel. The total frequencies of LoF alleles in taste receptors was calculated by adding together all LoF frequencies and were divided by the number of taste receptors using Excel. Then I compared these LoF frequencies among taste receptors, olfactory receptors and all genes in human genome. The calculation of overall average of human genes includes both LoF frequencies of taste and smell receptor genes.

### Multivariate analysis

Hierarchical clustering analysis was performed using the R 3.01 statistical software together with the Rcmdr package. I employed both Ward’s and median algorithms to configure the setting for clustering coefficient. Non-Hierarchical clustering algorithms was based on k-means approach. To separate confused data sets to make distinct classes, principal component analysis (PCA) was also performed on the matrices of spectra from 14 + 6 ethnic groups. Two-dimensional score plots and loading profiles of the principal components (PC 1 and 2) were applied to visualize the relative contribution of people’s taste preferences to the clustering of the different spectra.

### Evolutionary scenario of LoF variants in taste receptor genes

To estimate how much LoF events in taste receptor genes had occurred before and after the divergence from African origins, I compared alleles among individuals of various ethnic backgrounds Evidence of phylogeny could be based on significant differences in pair-wise comparisons between populations if two groups are significantly different (2-sample test for equality of proportions with continuity correction). The standard hypothesis test is *H*_0_: π_1_ = π_2_ against the alternative (two-sided) *H*_0_: π_1_ ≠ π_2_. The pairwise prop test can be used for testing the null that the proportions (probabilities of success) in two groups are the same. It is referred to as a z-test because the statistics looks like.


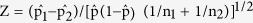


where pˆ = (p_1_ + p_2_)/(n_1_ + n_2_), and indices^1^,^2^ refers to the first and second line of the Table. In a two-way contingency Table where *H*_0_: p_1_ = p_2_, this should yield comparable results to the ordinary χ2 test.

### Estimates of allele age

Let *t*_*n,b*_ denote the age of a mutant having *b* copies in a sample of *n* genes, for 0 < *b* < *n*. Griffiths and Tavaré^60^ (see also Ref. 46) showed that the mean of *t*_*n,b*_ in a constant population can be obtained as,


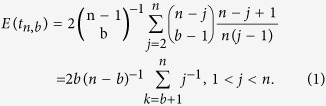


The formula for the expected square of the age, conditional on seeing *b* mutants in a sample of *n* genes is:


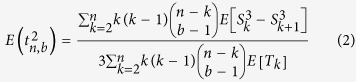


where 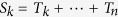, where *T*_*k*_ (*k* = *n*,…,2) is coalescent time which measures the time from *k* lineages to (*k* − 1) lineages and independent exponential random variables with means 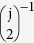.

The denominator of (2) is equal to:


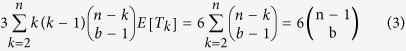


For the numerator of (2):


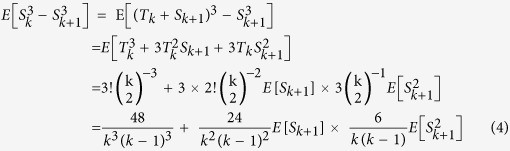


So


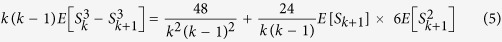


When 0 < *k* < *n*


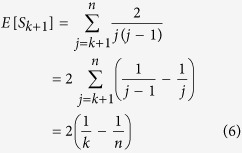


The variance of *t*_*n,b*_ can be obtained as,


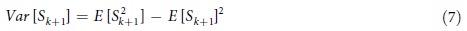


For all results reported in the manuscript, we assumed a generation time of 25 years.

## Additional Information

**How to cite this article**: Fujikura, K. Multiple loss-of-function variants of taste receptors in modern humans. *Sci. Rep.* **5**, 12349; doi: 10.1038/srep12349 (2015).

## Supplementary Material

Supplementary Information
