# Corrigendum: Multiple loss-of-function variants of taste receptors in modern humans

**DOI:** 10.1038/srep20741

**Published:** 2016-02-08

**Authors:** Kohei Fujikura

Scientific Reports 5: Article number: 1234910.1038/srep12349; published online: 08262015; updated: 02082016.

The original version of this Article contained an error in the spelling of the author Kohei Fujikura, which was incorrectly given as K. Fujikura. This has now been corrected in both the PDF and HTML versions of the Article.

