# Supplementary material for: Multiple loss-of-function variants of taste receptors in modern humans
Source: Sci Rep. 2015 Aug 26;5:12349. doi: 10.1038/srep12349 (PMC4549710; doi:10.1038/srep12349)
Supplement: Supplementary Information [file srep12349-s1.pdf]

Supplementary Information

Multiple loss-of-function variants of taste receptors in modern humans

Kohei Fujikura[1]*

1 Kobe University School of Medicine, 7-5-1, Kusunoki-cho, Chuo-ku, Kobe 650-0017, Japan

***Correspondence should be addressed to Kohei Fujikura.

Telephone: +81-90-3906-9772

Email: kofujikura@gmail.com

Figures S1-S4

Tables S1-S4

Note 1

I analyzed 45 taste-buds enriched genes (*C6orf15*, *CXCL14*, *SLC35F1*, *PDE1C*, *MMP7*, *GFRA3*, *AVIL*, *ZNF483*, *KCNE3*, *PLCD4*, *CAPN9*, *ASCL1*, *LOC644139*, *COL9A2*, *IGF1*, *CPE*, *PTPRD*, *LOC253012*, *TOX3*, *Hs. 164557*, *TUBB2B*, *ESPN*, *SATB2*, *MCTP1*, *SCN3A*, *TGFB2*, *DCLK1*, *SCG5*, *KRT20*, *IL8*, *PROX1*, *INSM1*, *SYT1*, *NRCAM*, *SEZ6L*, *KIAA1324*, *SCD5*, *SEMA3D*, *TMEM163*, *CDH2*, *NRXN3*, *CCL2*, *SHH*, *BAMBI*, *JAKMIP2*, *GNB3*) using NHLBI and 1000 genome sequences. 51.4% (18/35) of taste receptors are frequently lost in modern humans (Figure 2A). In contrast only 4.4% (2/45) of taste-buds enriched genes were frequent target of LoF mutations.

Note 2

The genotype accuracy of 1000 Genomes was previously estimated at 97.4% (20,687/21,235) by comparing with the HapMap genotype calls. The validity of a part of the NHLBI datasets were assessed by NHLBI using Sanger sequencing (novel singleton variants, 143/145 (99%); novel non-singleton variants 316/323 (98%)).

Figure S1

The mutation rates plotted against the chromosome positions (Chr 16) of *PKD1L3*

The allele frequency is shown together with the positions and orientations of genes (NCBI Build 37). Two ethnic groups (European and African) are compared to each other with respect to mutational patterns.

fig. S1

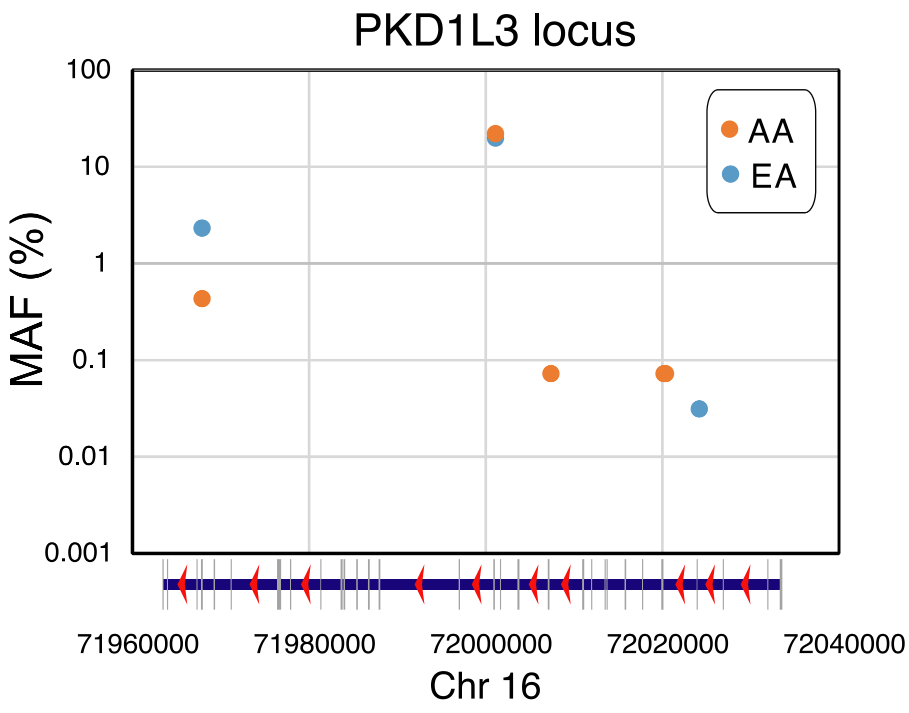

PKD1L3 locus
100
10
1
0.1
0.01
0.001
MAF (%)
AA
EA
71960000
71980000
72000000
72020000
72040000
Chr 16

Figure S2

Section of 20x20 scatterplot matrix of 20 genetic variants of taste related genes.

rs7904983, rs34789740 and rs41324347 are highly correlated with each other as African common allele while rs77837442, rs140193828 and rs147505098 are in European. rs4788587 and rs2708381 are correlated in Asian population.

fig. S2

Cluster1 +	Cluster2 ○	Cluster3 △
AA	ASN	EA
AFR	CHB	AMR
ASW	CHS	CEU
LWK	JPT	CLM
YRI		EUR
		FIN
		GBR
		IBS
		MXL
		PUR
		TSI

African common allele

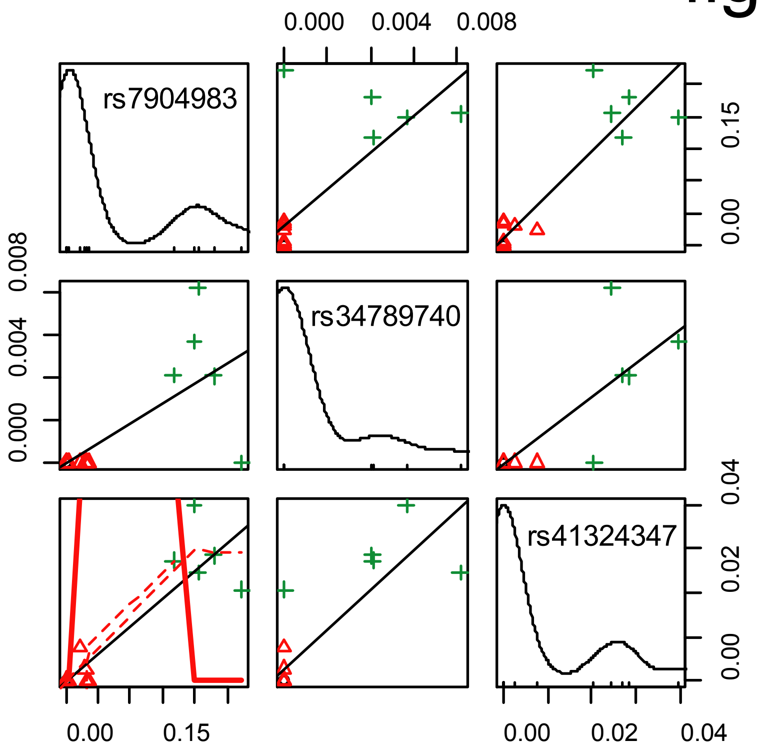

European common allele

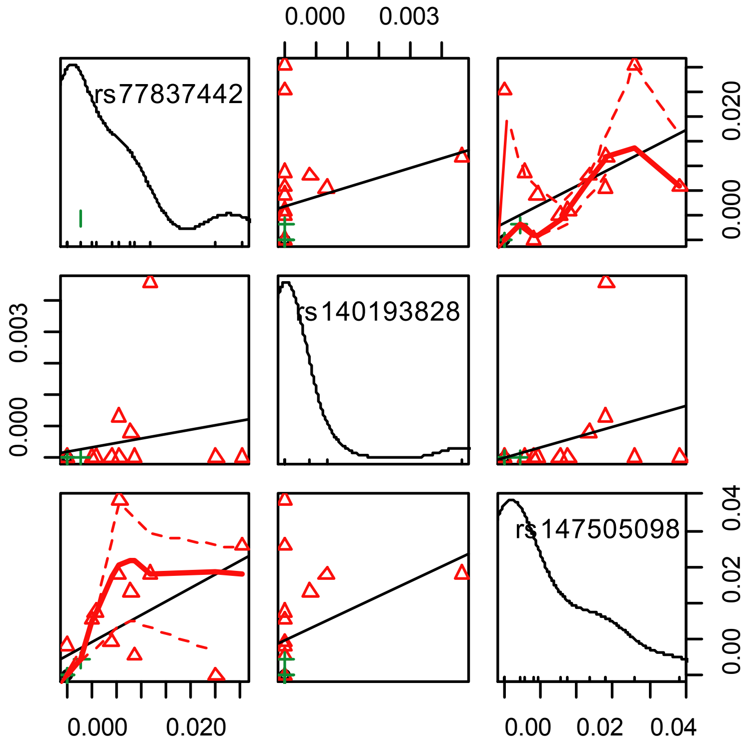

Asian common allele

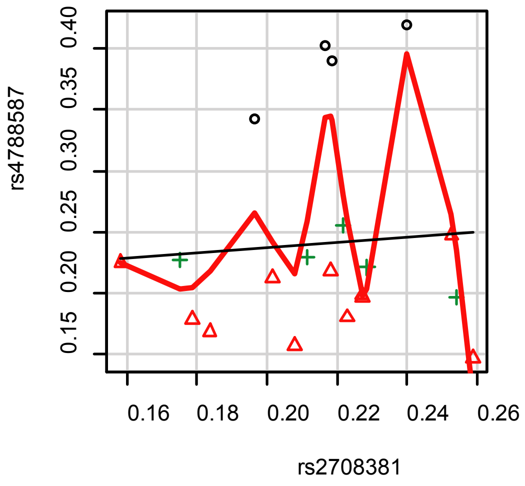

Figure S3

Dendrogram of 14 + 6 ethnic groups obtained with hierarchical median method.

Vertical axis correspond to squared Euclidean distance. Abbreviations are as follows: AFR, African; AMR, Ad mixed American; ASN, East Asian; ASW, American's of African Ancestry in SW; CEU, Utah Residents (CEPH) with Northern and Western European ancestry ; CHB, Han Chinese in Beijing; CHS, Southern Han Chinese; CLM, Colombian from Medellian; EUR, European; FIN, Finnish in Finland; GBR, British in England; IBS, Iberian population in Spain; JPT, Japanese in Tokyo; LWK, Luhya in Webuye; MXL, Mexican ancestry from Los Angeles; PUR, Puerto Rico from Puerto Rica; TSI, Toscani in Italia; YRI, Yorba in Ibadan.

fig. S3

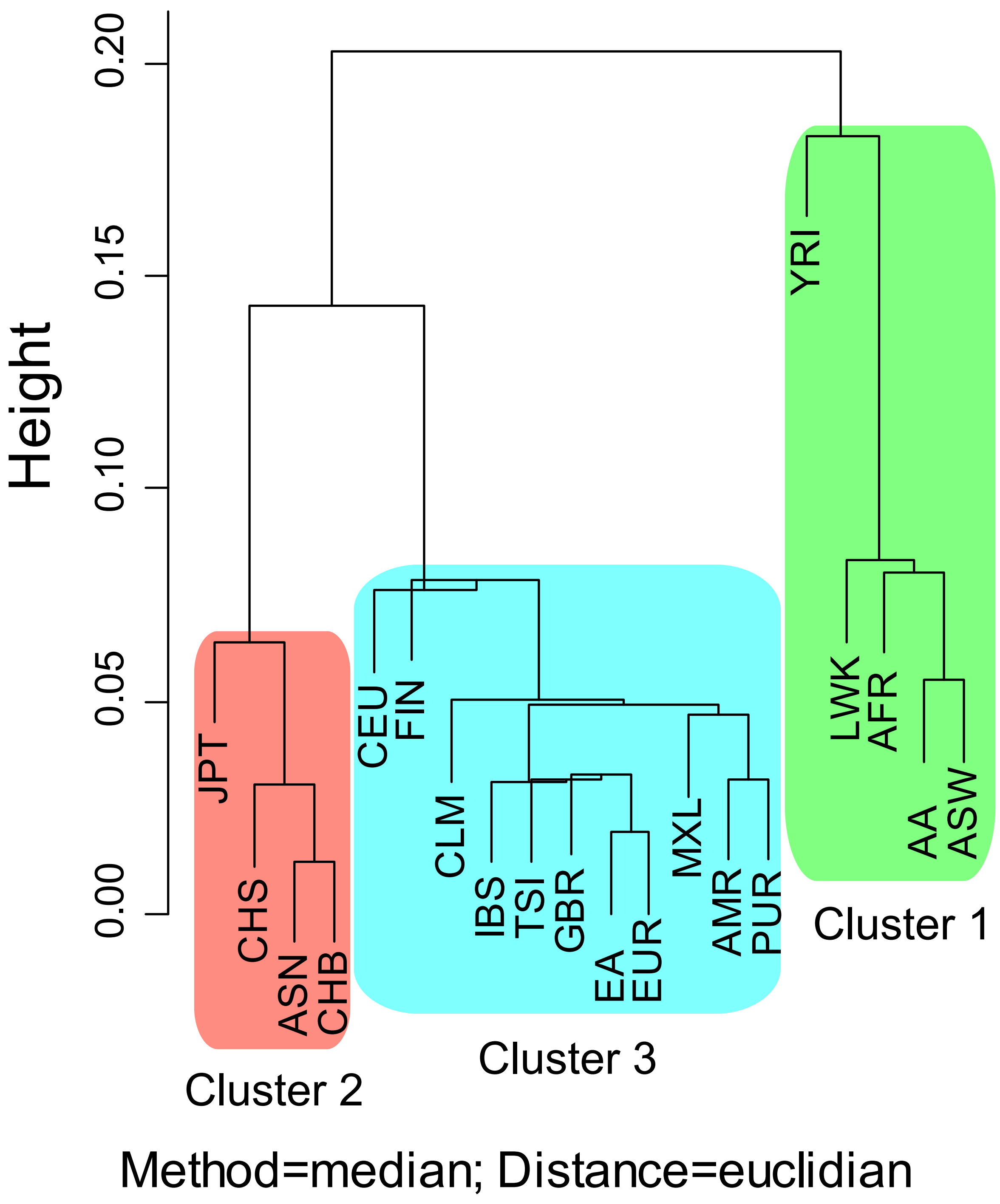
Height
0.00
0.05
0.10
0.15
0.20
JPT
CHS
ASN
CHB
CEU
FIN
CLM
IBS
TSI
GBR
EA
EUR
MXL
AMR
PUR
YRI
LWK
AFR
AA
ASW
Cluster 1
Cluster 2
Cluster 3
Method=median; Distance=euclidian

Figure S4

Spatial graphs of 14 + 6 ethnic groups based on 20x20 scatterplot matrix.

Three axes correspond to the allele frequency of rs4788587, rs7904983 and rs77837442. Orange, green and blue spheres indicate three clusters derived from Hierarchical and non-Hierarchical clustering methods.

fig. S4

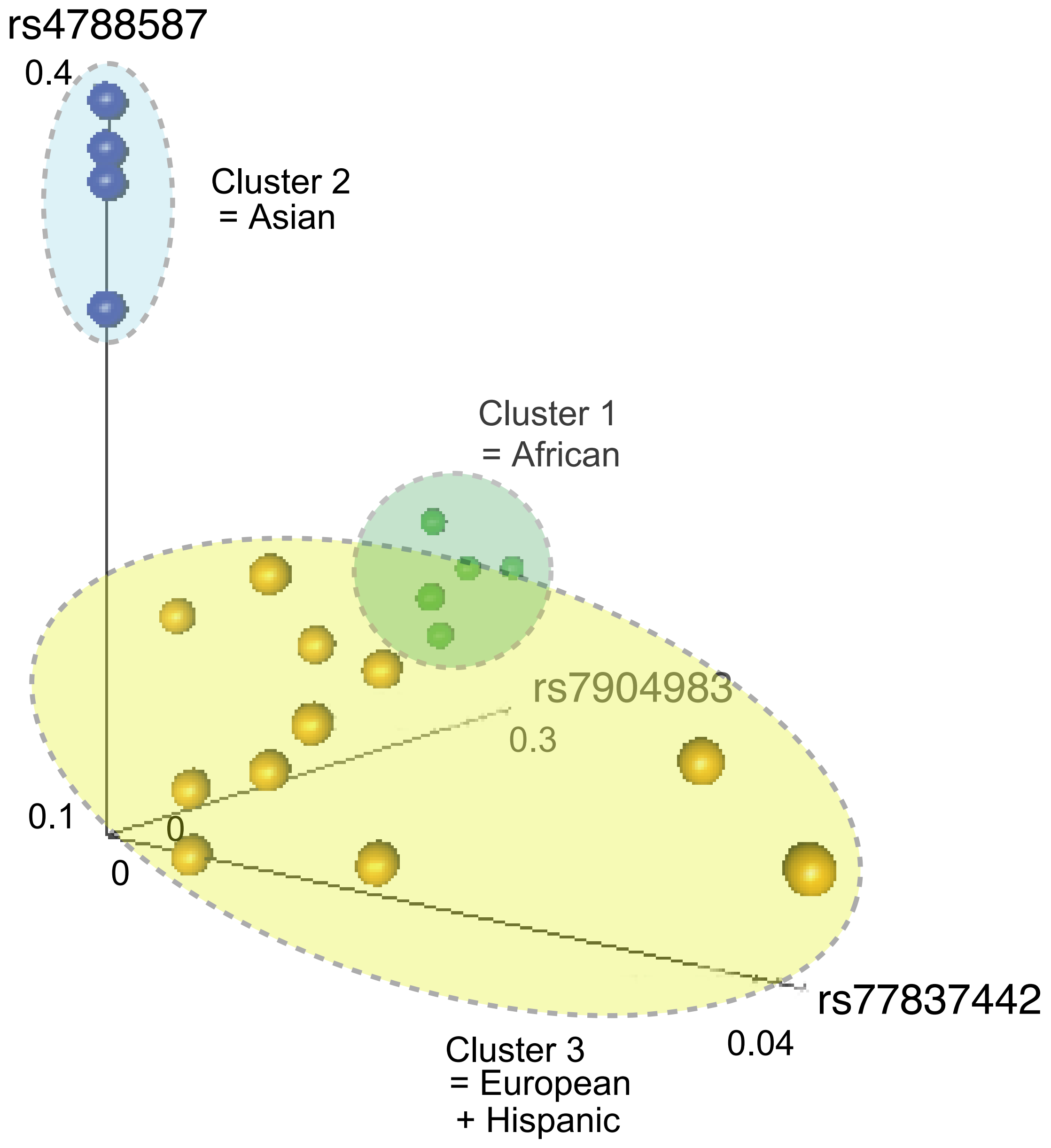
rs4788587
0.4
Cluster 2
= Asian
Cluster 1
= African
rs7904983
0.3
0.1
0
0
rs77837442
0.04
Cluster 3
= European
+ Hispanic

Table S1

Target gene lists analyzed in this study and representative reference lists.

Table S1

Gene Name	References
TAS1R1	(Nelson et al. 2002 [1]; Li et al. 2002 [2]; Chen et al. 2009 [3])
TAS1R2	(Li et al. 2002 [2]; Nelson et al. 2002 [4]; Eny et al. 2010 [5])
TAS1R3	(Nelson et al. 2002 [1]; Li et al. 2002 [2]; Nelson et al. 2002 [4])
TAS2R1	(Meyerhof et al. 2010 [6]; Kuhn et al. 2010 [7])
TAS2R3	(Meyerhof et al. 2010 [6]; Kuhn et al. 2010 [7])
TAS2R4	(Meyerhof et al. 2010 [6]; Kuhn et al. 2010 [7]; Soares et al. 2013 [8])
TAS2R5	(Meyerhof et al. 2010 [6]; Kuhn et al. 2010 [7]; Soares et al. 2013 [8])
TAS2R7	(Meyerhof et al. 2010 [6]; Kuhn et al. 2010 [7]; Soares et al. 2013 [8])
TAS2R8	(Meyerhof et al. 2010 [6]; Kuhn et al. 2010 [7]; Ueno et al. 2011 [9])
TAS2R9	(Meyerhof et al. 2010 [6]; Kuhn et al. 2010 [7]; Allen et al. 2013 [10])
TAS2R10	(Meyerhof et al. 2010 [6]; Kuhn et al. 2010 [7]; Born et al. 2013 [11])
TAS2R13	(Meyerhof et al. 2010 [6]; Kuhn et al. 2010 [7]; Dotson et al. 2013 [12])
TAS2R14	(Meyerhof et al. 2010 [6]; Kuhn et al. 2010 [7]; Roland et al. 2013 [13])
TAS2R16	(Meyerhof et al. 2010 [6]; Kuhn et al. 2010 [7]; Bofe et al. 2002 [14])
TAS2R19	(Meyerhof et al. 2010 [6]; Kuhn et al. 2010 [7]; Reed et al. 2010 [15])
TAS2R20	(Meyerhof et al. 2010 [6]; Kuhn et al. 2010 [7])
TAS2R30	(Meyerhof et al. 2010 [6]; Kuhn et al. 2010 [7])
TAS2R31	(Meyerhof et al. 2010 [6]; Kuhn et al. 2010 [7]; Allen et al. 2013 [10];
	Brockhoff et al. 2010 [16]; Roundnitzky et al. 2011 [17])
TAS2R38	(Meyerhof et al. 2010 [6]; Kuhn et al. 2010 [7]; Reed et al. 2010 [15];
	Bufe et al. 2005 [18]; Behrens et al. 2012 [19]; Knaapila et al. 2012 [20])
TAS2R39	(Meyerhof et al. 2010 [6]; Kuhn et al. 2010 [7]; Ueno et al. 2011 [9])
TAS2R40	(Meyerhof et al. 2010 [6]; Kuhn et al. 2010 [7])
TAS2R41	(Meyerhof et al. 2010 [6]; Kuhn et al. 2010 [7]; Thalmann et al. 2013 [21])
TAS2R42	(Meyerhof et al. 2010 [6]; Kuhn et al. 2010 [7])
TAS2R43	(Meyerhof et al. 2010 [6]; Kuhn et al. 2010 [7]; Brockhoff et al. 2010 [16];
	Roundnitzky et al. 2011 [17])
TAS2R45	(Meyerhof et al. 2010 [6]; Kuhn et al. 2010 [7])
TAS2R46	(Meyerhof et al. 2010 [6]; Kuhn et al. 2010 [7]; Brockhoff et al. 2010 [16];
	Roundnitzky et al. 2011 [17]; Brockhoff et al. 2010 [22])
TAS2R50	(Meyerhof et al. 2010 [6]; Kuhn et al. 2010 [7]; Knaapila et al. 2012 [20];
	Behrens et al. 2009 [23])
TAS2R60	(Meyerhof et al. 2010 [6]; Kuhn et al. 2010 [7])
PKD1L3	(Li et al. 2003 [24]; Ishimaru et al. 2006 [25]; Huang et al. 2006 [26];
	LopezJimenez et al. 2006 [27])
PKD2L1	(Ishimaru et al. 2006 [25]; Huang et al. 2006 [26]; LopezJimenez et al. 2006 [27])
CD36	(Laugerette et al. 2005 [28]; Sclafani et al. 2007 [29]; Simons et al. 2011 [30])
ENaCa	(Chandrashekar et al. 2010 [31])
ENaCd	(Stahler et al. 2008 [32]; Huque et al. 2009 [33]; Ji et al. 2012 [34])
HCN1	(Stevens et al. 2001 [35])
HCN4	(Stevens et al. 2001 [35]; Gao et al. 2009 [36])

A full reference list is as follows:

1. Nelson G, Chandrashekar J, Hoon MA, Feng L, Zhao G, Ryba NJ, Zuker CS. An amino-acid taste receptor. Nature. 2002 416(6877):199-202.

2. Li X, Staszewski L, Xu H, Durick K, Zoller M, Adler E. Human receptors for sweet and umami taste. Proc Natl Acad Sci U S A. 2002 99(7):4692-6.

3. Chen QY, Alarcon S, Tharp A, Ahmed OM, Estrella NL, Greene TA, Rucker J, Breslin PA. Perceptual variation in umami taste and polymorphisms in TAS1R taste receptor genes. Am J Clin Nutr. 2009 90(3):770S-779S.

4. Nelson G, Hoon MA, Chandrashekar J, Zhang Y, Ryba NJ, Zuker CS. Mammalian sweet taste receptors. Cell. 2001 106(3):381-90.

5. Eny KM, Wolever TM, Corey PN, El-Sohemy A. Genetic variation in TAS1R2 (Ile191Val) is associated with consumption of sugars in overweight and obese individuals in 2 distinct populations. Am J Clin Nutr. 2010 92(6):1501-10.

6. Meyerhof W, Batram C, Kuhn C, Brockhoff A, Chudoba E, Bufe B, Appendino G, Behrens M. The molecular receptive ranges of human TAS2R bitter taste receptors. Chem Senses. 2010 35(2):157-70.

7. Kuhn C, Bufe B, Batram C, Meyerhof W. Oligomerization of TAS2R bitter taste receptors. Chem Senses. 2010 35(5):395-406.

8. Soares S, Kohl S, Thalmann S, Mateus N, Meyerhof W, De Freitas V. Different phenolic compounds activate distinct human bitter taste receptors. J Agric Food Chem. 2013 61(7):1525-33.

9. Ueno Y, Sakurai T, Okada S, Abe K, Misaka T. Human bitter taste receptors hTAS2R8 and hTAS2R39 with differential functions to recognize bitter peptides. Biosci Biotechnol Biochem. 2011 75(6):1188-90.

10. Allen AL, McGeary JE, Knopik VS, Hayes JE. Bitterness of the non-nutritive sweetener acesulfame potassium varies with polymorphisms inTAS2R9 and TAS2R31. Chem Senses. 2013 38(5):379-89.

11. Born S, Levit A, Niv MY, Meyerhof W, Behrens M. The human bitter taste receptor TAS2R10 is tailored to accommodate numerous diverse ligands. J Neurosci. 2013 33(1):201-13.

12. Dotson CD, Wallace MR, Bartoshuk LM, Logan HL. Variation in the gene TAS2R13 is associated with differences in alcohol consumption in patients with head and neck cancer. Chem Senses. 2012 37(8):737-44.

13. Roland WS, van Buren L, Gruppen H, Driesse M, Gouka RJ, Smit G, Vincken JP. Bitter Taste Receptor Activation by Flavonoids and Isoflavonoids: Modeled Structural Requirements for Activation of hTAS2R14 and hTAS2R39. J Agric Food Chem. 2013

14. Bufe B, Hofmann T, Krautwurst D, Raguse JD, Meyerhof W. The human TAS2R16 receptor mediates bitter taste in response to beta-glucopyranosides. Nat Genet. 2002 32(3):397-401.

15. Reed DR, Zhu G, Breslin PA, Duke FF, Henders AK, Campbell MJ, Montgomery GW, Medland SE, Martin NG, Wright MJ. The perception of quinine taste intensity is associated with common genetic variants in a bitter receptor cluster on chromosome 12. Hum Mol Genet. 2010 19(21):4278-85.

16. Brockhoff A, Behrens M, Niv MY, Meyerhof W. Structural requirements of bitter taste receptor activation. Proc Natl Acad Sci U S A. 2010 107(24):11110-5.

17. Roudnitzky N, Bufe B, Thalmann S, Kuhn C, Gunn HC, Xing C, Crider BP, Behrens M, Meyerhof W, Wooding SP. Genomic, genetic and functional dissection of bitter taste responses to artificial sweeteners. Hum Mol Genet. 2011 20(17):3437-49.

18. Bufe B, Breslin PA, Kuhn C, Reed DR, Tharp CD, Slack JP, Kim UK, Drayna D, Meyerhof W. The molecular basis of individual differences in phenylthiocarbamide and propylthiouracil bitterness perception. Curr Biol. 2005 15(4):322-7.

19. Behrens M, Born S, Redel U, Voigt N, Schuh V, Raguse JD, Meyerhof W. Immunohistochemical detection of TAS2R38 protein in human taste cells. PLoS One. 2012 7(7):e40304.

20. Knaapila A, Hwang LD, Lysenko A, Duke FF, Fesi B, Khoshnevisan A, James RS, Wysocki CJ, Rhyu M, Tordoff MG, Bachmanov AA, Mura E, Nagai H, Reed DR. Genetic analysis of chemosensory traits in human twins. Chem Senses. 2012 37(9):869-81.

21. Thalmann S, Behrens M, Meyerhof W. Major haplotypes of the human bitter taste receptor TAS2R41 encode functional receptors for chloramphenicol. Biochem Biophys Res Commun. 2013 435(2):267-73.

22. Brockhoff A, Behrens M, Massarotti A, Appendino G, Meyerhof W. Broad tuning of the human bitter taste receptor hTAS2R46 to various sesquiterpene lactones, clerodane and labdane diterpenoids, strychnine, and denatonium. J Agric Food Chem. 2007 55: 6236–6243.

23. Behrens M, Brockhoff A, Batram C, Kuhn C, Appendino G, Meyerhof W. The human bitter taste receptor hTAS2R50 is activated by the two natural bitter terpenoids andrographolide and amarogentin. J Agric Food Chem 2009 57: 9860–9866.

24. Li A, Tian X, Sung SW, Somlo S. Identification of two novel polycystic kidney disease-1-like genes in human and mouse genomes. Genomics. 2003 81(6):596-608.

25. Ishimaru Y, Inada H, Kubota M, Zhuang H, Tominaga M, Matsunami H. Transient receptor potential family members PKD1L3 and PKD2L1 form a candidate sour taste receptor. Proc Natl Acad Sci U S A. 2006 103(33):12569-74.

26. Huang AL, Chen X, Hoon MA, Chandrashekar J, Guo W, Tränkner D, Ryba NJ, Zuker CS. The cells and logic for mammalian sour taste detection. Nature. 2006 442(7105):934-8.

27. LopezJimenez ND, Cavenagh MM, Sainz E, Cruz-Ithier MA, Battey JF, Sullivan SL. Two members of the TRPP family of ion channels, Pkd1l3 and Pkd2l1, are co-expressed in a subset of taste receptor cells. J Neurochem. 2006 98(1):68-77.

28. Laugerette F, Passilly-Degrace P, Patris B, Niot I, Febbraio M, Montmayeur JP, Besnard P. CD36 involvement in orosensory detection of dietary lipids, spontaneous fat preference, and digestive secretions. J Clin Invest. 2005 115(11):3177-84.

29. Sclafani A, Ackroff K, Abumrad NA. CD36 gene deletion reduces fat preference and intake but not post-oral fat conditioning in mice. Am J Physiol Regul Integr Comp Physiol. 2007 293(5):R1823-32.

30. Simons PJ, Kummer JA, Luiken JJ, Boon L. Apical CD36 immunolocalization in human and porcine taste buds from circumvallate and foliate papillae. Acta Histochem. 2011 113(8):839-43.

31. Chandrashekar J, Kuhn C, Oka Y, Yarmolinsky DA, Hummler E, Ryba NJ, Zuker CS. The cells and peripheral representation of sodium taste in mice. Nature. 2010 464(7286):297-301.

32.Stahler, F., Riedel, K., Demgensky, S., Neumann, K., Dunkel, A., Taubert, A., Raab, B., Behrens, M., Raguse, J.D., Hofman, T., Meyerhof, W. (2008): A role of the epithelial sodium channel in human salt taste transduction? Chem Percept 1 (1), 78 - 90

33. Huque T, Cowart BJ, Dankulich-Nagrudny L, Pribitkin EA, Bayley DL, Spielman AI, Feldman RS, Mackler SA, Brand JG. Sour ageusia in two individuals implicates ion channels of the ASIC and PKD families in human sour taste perception at the anterior tongue. PLoS One. 2009 4(10):e7347.

34. Ji HL, Zhao RZ, Chen ZX, Shetty S, Idell S, Matalon S.δ ENaC: a novel divergent amiloride-inhibitable sodium channel. Am J Physiol Lung Cell Mol Physiol. 2012 303(12):L1013-26.

35. Stevens DR, Seifert R, Bufe B, Müller F, Kremmer E, Gauss R, Meyerhof W, Kaupp UB, Lindemann B. Hyperpolarization-activated channels HCN1 and HCN4 mediate responses to sour stimuli. Nature. 2001 413(6856):631-5.

36. Gao N, Lu M, Echeverri F, Laita B, Kalabat D, Williams ME, Hevezi P, Zlotnik A, Moyer BD. Voltage-gated sodium channels in taste bud cells. BMC Neurosci. 2009 10:20.

Table S2

Summary for allele frequency of LoF variants of taste receptor genes in representative six data sets.

Table S2

Tastant quality	Gene	#base(NCBI.37)	Chromosome	rsID	Mutation type	Alleles	NHLBI EuropeanAmerican	NHLBI AfricanAmerican	1000GENOMES AFR	1000GENOMES AMR	1000GENOMES ASN	1000GENOMES EUR	ALL
Sour	PKD1L3	NM_181536.1	Ch16	rs147505098	stop-gained	A/G	74/3182 (0.0233)	6/1384 (0.0043)	0/492 (0)	2/362 (0.0055)	0/572 (0)	21/758 (0.0277)	103/6750 (0.0153)
				rs4788587	stop-gained	A/G	632/3182 (0.1986)	306/1384 (0.2211)	113/492 (0.2297)	77/362 (0.2127)	223/577 (0.3865)	137/758 (0.1807)	1488/6755 (0.2203)
	PKD2L1	NM_016112.2	Ch10	rs7904983	stop-gained	A/G	10/8600 (0.0011)	735/4406 (0.1668)	113/492 (0.2297)	11/362 (0.0304)	0/572 (0)	1/758 (0.0013)	870/15190 (0.0573)
Umami	TAS1R1	NM_138697.3	Ch1	rs149094445	stop-gained	T/C	0/8592 (0)	26/4402 (0.0059)	1/492 (0.0020)	0/362 (0)	0/572 (0)	0/758 (0)	27/15178 (0.0018)
				rs140193828	stop-gained	A/G	6/7604 (0.0008)	0/3880 (0)	0/492 (0)	0/362 (0)	0/572 (0)	1/758 (0.0013)	7/13668 (0.0005)
				rs115030111	stop-gained	A/G	1/8600 (0.0001)	15/4406 (0.0034)	2/492 (0.0041)	0/362 (0)	0/572 (0)	0/758 (0)	18/15190 (0.0012)
Sweet	TAS1R3	NM_152228.1	Ch1	rs141631187	5'splice	A/G	0/7006 (0)	2/3730 (0.0005)	0/492 (0)	0/362 (0)	0/572 (0)	0/758 (0)	2/12920 (0.0002)
Bitter	TAS2R3	NM_016943.2	Ch7	rs138466830	stop-gained	A/G	19/8600 (0.0022)	4/4406 (0.0009)	0/492 (0)	0/362 (0)	0/572 (0)	2/758 (0.0026)	25/15190 (0.0016)
	TAS2R7	NM_023919.2	Ch12	rs150192473	stop-gained	A/G	1/8600 (0.0001)	2/4406 (0.0005)	1/492 (0.0020)	12/362 (0.0331)	0/572 (0)	0/758 (0)	16/15190 (0.0011)
	TAS2R8	NM_023918.1	Ch12	rs41324347	stop-gained	A/C	1/8594 (0.0001)	120/4406 (0.0272)	14/492 (0.0285)	1/362 (0.0028)	0/572 (0)	0/758 (0)	136/15184 (0.0090)
	TAS2R14	NM_023922.1	Ch12	rs34789740	stop-gained	A/G	0/8600 (0)	18/4406 (0.0041)	2/492 (0.0041)	0/362 (0)	0/572 (0)	0/758 (0)	20/15190 (0.0013)
	TAS2R19	NM_176888.1	Ch12	rs77837442	stop-gained	T/C	113/8600 (0.0131)	13/4406 (0.0030)	0/492 (0)	5/362 (0.0138)	0/572 (0)	8/758 (0.0106)	139/15190 (0.0092)
	TAS2R20	NM_176889.2	Ch12	rs116400924	stop-gained	T/C	1/8600 (0.0001)	49/4406 (0.0111)	6/492 (0.0122)	0/362 (0)	0/572 (0)	0/758 (0)	56/15190 (0.0037)
	TAS2R40	NM_176882.1	Ch7	rs145138257	stop-gained	T/C	8/8258 (0.0010)	0/3804 (0)	0/492 (0)	0/362 (0)	0/572 (0)	2/758 (0.0026)	10/14246 (0.0007)
	TAS2R41	NM_176883.2	Ch7	rs201255995	stop-gained	A/G	0/8362 (0)	7/3992 (0.0018)	0/492 (0)	0/362 (0)	0/572 (0)	0/758 (0)	7/12354 (0.0006)
	TAS2R42	NM_181429.1	Ch12	rs141369006	stop-gained	T/C	0/8600 (0)	11/4406 (0.0025)	1/492 (0.0020)	1/362 (0.0028)	0/572 (0)	0/758 (0)	13/15190 (0.0009)
	TAS2R43	NM_176884.2	Ch12	rs113710768	stop-gained	T/C	1542/8156 (0.1891)	399/3776 (0.1057)					1941/11932 (0.1627)
	TAS2R46	NM_176887.2	Ch12	rs150894148	stop-gained	A/G	0/8484 (0)	38/4150 (0.0092)	7/492 (0.0142)	0/362 (0)	0/572 (0)	0/758 (0)	45/14818 (0.0030)
				rs2708381	stop-gained	T/C	1953/8600 (0.2271)	1007/4406 (0.2286)	104/492 (0.2114)	73/362 (0.2017)	125/572 (0.2185)	169/572 (0.2955)	3431/15190 (0.2259)
	TAS2R60	NM_177437.1	Ch7	rs138984388	stop-gained	G/C	1/8600 (0.0001)	9/4406 (0.0020)	1/492 (0.0020)	0/362 (0)	0/572 (0)	0/758 (0)	11/15190 (0.0007)
Sodium	SCNN1D	NM_001130413.3	Ch1	rs148588796	stop-gained	T/C	0/8578 (0)	8/4396 (0.0018)	2/492 (0.0041)	0/362 (0)	0/572 (0)	0/758 (0)	10/15158 (0.0007)
Fat	CD36	NM_000072.3	Ch7	rs114881528	5'splice	A/G	0/8600 (0)	3/4406 (0.0007)	1/492 (0.0020)	0/362 (0)	0/572 (0)	0/758 (0)	4/15190 (0.0003)
				rs3211893	5'splice	C/T	0/8600 (0)	22/4406 (0.0050)	8/492 (0.0020)	1/362 (0.0028)	0/572 (0)	0/758 (0)	31/15190 (0.0020)
				rs3211938	stop-gained	G/T	2/8600 (0.0002)	361/4406 (0.0819)	72/492 (0.1463)	0/362 (0)	0/572 (0)	0/758 (0)	363/13006 (0.0279)
				rs56381858	stop-gained	G/T	2/8592 (0.0002)	0/4406 (0)	0/492 (0)	0/362 (0)	0/572 (0)	0/758 (0)	2/15182 (0.0001)

Table S3

Summary for allele frequency of LoF variants of taste receptor genes in 14+6 (1000 genome+NHLBI) ethnic groups.

Table S3

Gene	#base(NCBI.37)	rsID	Alleles	AFR	AMR	ASN	ASW	CEU	CHB	CHS	CLM	EUR
PKD1L3	NM_181536.1	rs147505098	A/G	246 (GIG)	2 (AIG) / 179 (GIG)	286 (GIG)	61 (GIG)	3 (AIG) / 82 (GIG)	97 (GIG)	100 (GIG)	1 (AIG) / 59 (GIG)	21 (AIG) / 358 (GIG)
		rs4788587	A/G	12 (AIA) / 89 (AIG) / 145 (GIG)	9 (AIA) / 59 (AIG) / 113 (GIG)	44 (AIA) / 135 (AIG) / 107	3 (AIA) / 18 (AIG) / 40 (GIG)	2 (AIA) / 21 (AIG) / 62 (GIG)	19 (AIA) / 40 (AIG) / 38 (GIG)	15 (AIA) / 54 (AIG) / 31 (GIG)	1 (AIA) / 25 (AIG) / 34 (GIG)	16 (AIA) / 105 (AIG) / 258
PKD2L1	NM_016112.2	rs7904983	A/G	12 (AIA) / 89 (AIG) / 145 (GIG)	11 (AIG) / 170 (GIG)	286 (GIG)	2 (AIA) / 21 (AIG) / 38 (GIG)	85 (GIG)	97 (GIG)	100 (GIG)	4 (AIG) / 56 (GIG)	1 (AIG) / 378 (GIG)
TAS1R1	NM_138697.3	rs149094445	T/C	245 (CIC) / 1 (CIT)	181 (CIC)	286 (CIC)	60 (CIC) / 1 (CIT)	85 (CIC)	97 (CIC)	100 (CIC)	60 (CIC)	379 (CIC)
		rs140193828	A/G	246 (GIG)	181 (GIG)	286 (GIG)	61 (GIG)	85 (GIG)	97 (GIG)	100 (GIG)	60 (GIG)	1 (AIG) / 378 (GIG)
		rs115030111	A/G	2 (AIG) / 244 (GIG)	181 (GIG)	286 (GIG)	1 (AIG) / 60 (GIG)	85 (GIG)	97 (GIG)	100 (GIG)	60 (GIG)	379 (GIG)
TAS1R3	NM_152228.1	rs141631187	A/G	246 (GIG)	181 (GIG)	286 (GIG)						379 (GIG)
TAS2R3	NM_016943.2	rs138466830	A/G	246 (GIG)	181 (GIG)	286 (GIG)	61 (GIG)	2 (AIG) / 83 (GIG)	97 (GIG)	100 (GIG)	60 (GIG)	2 (AIG) / 377 (GIG)
TAS2R7	NM_023919.2	rs150192473	A/G	1 (AIG) / 245 (GIG)	1 (AIA) / 10 (AIG) / 170 (GIG)	286 (GIG)	1 (AIG) / 60 (GIG)	85 (GIG)	97 (GIG)	100 (GIG)	1 (AIA) / 1 (AIG) / 58 (GIG)	379 (GIG)
TAS2R8	NM_023918.1	rs41324347	A/C	1 (AIA) / 12 (AIC) / 233 (CIC)	1 (AIC) / 180 (CIC)	286 (CIC)	3 (AIC) / 58 (CIC)	85 (CIC)	97 (CIC)	100 (CIC)	60 (CIC)	379 (CIC)
TAS2R14	NM_023922.1	rs34789740	A/G	2 (AIG) / 244 (GIG)	181 (GIG)	286 (GIG)	1 (AIG) / 60 (GIG)	85 (GIG)	97 (GIG)	100 (GIG)	60 (GIG)	379 (GIG)
TAS2R19	NM_176888.1	rs77837442	T/C	246 (CIC)	176 (CIC) / 5 (CIT)	286 (CIC)	61 (CIC)	84 (CIC) / 1 (CIT)	97 (CIC)	100 (CIC)	60 (CIC)	371 (CIC) / 8 (CIT)
TAS2R20	NM_176889.2	rs116400924	T/C	240 (CIC) / 6 (CIT)	181 (CIC)	286 (CIC)	60 (CIC) / 1 (CIT)	85 (CIC)	97 (CIC)	100 (CIC)	60 (CIC)	379 (CIC)
TAS2R40	NM_176882.1	rs145138257	T/C	246 (CIC)	181 (CIC)	286 (CIC)	61 (CIC)	85 (CIC)	97 (CIC)	100 (CIC)	60 (CIC)	377 (CIC) / 2 (CIT)
TAS2R41	NM_176883.2	rs201235995	A/G	246 (GIG)	181 (GIG)	286 (GIG)						379 (GIG)
TAS2R42	NM_181429.1	rs141369006	T/C	245 (CIC) / 1 (CIT)	180 (CIC) / 1 (CIT)	286 (CIC)	61 (CIC)	85 (CIC)	97 (CIC)	100 (CIC)	60 (CIC)	379 (CIC)
TAS2R43	NM_176884.2	rs113710768	T/C									
TAS2R46	NM_176887.2	rs150894148	A/G	239 (GIG) / 7 (GIA)	181 (GIG)	286 (GIG)						379 (GIG)
		rs2708381	T/C	152 (CIC) / 84 (CIT) / 10 (TIT)	113 (CIC) / 63 (CIT) / 5 (TIT)	176 (CIC) / 95 (CIT) / 15 (TIT)	34 (CIC) / 23 (CIT) / 4 (TIT)	48 (CIC) / 30 (CIT) / 7 (TIT)	59 (CIC) / 34 (CIT) / 4 (TIT)	59 (CIC) / 34 (CIT) / 7 (TIT)	43 (CIC) / 15 (CIT) / 2 (TIT)	231 (CIC) / 127 (CIT) / 21 (TIT)
TAS2R60	NM_177437.1	rs138984388	G/C	245 (CIC) / 1 (CIG)	181 (CIC)	286 (CIC)	60 (CIC) / 1 (CIG)	85 (CIC)	97 (CIC)	100 (CIC)	60 (CIC)	379 (CIC)
SCNN1D	NM_001130413.3	rs148588796	T/C	244 (CIC) / 2 (CIT)	181 (CIC)	286 (CIC)	60 (CIC) / 1 (CIT)	85 (CIC)	97 (CIC)	100 (CIC)	60 (CIC)	379 (CIC)
CD36	NM_000072.3	rs114881528	A/G	1 (AIG) / 245 (GIG)	181 (GIG)	286 (GIG)	61 (GIG)	85 (GIG)	97 (GIG)	100 (GIG)	60 (GIG)	379 (GIG)
		rs3211893	C/T	8 (CIT) / 238 (TIT)	1 (CIT) / 180 (TIT)	286 (TIT)	1 (CIT) / 60 (TIT)	85 (TIT)	97 (TIT)	100 (TIT)	1 (CIT) / 59 (TIT)	379 (TIT)
		rs3211938	G/T	9 (GIG) / 54 (GIT) / 183 (TIT)	181 (TIT)	286 (TIT)	1 (GIG) / 7 (GIT) / 53 (TIT)	85 (TIT)	97 (TIT)	100 (TIT)	60 (TIT)	379 (TIT)
		rs56381858	G/T	246 (GIG)	181 (GIG)	286 (GIG)						379 (GIG)

Gene	rsID	FIN	GBR	IBS	JPT	LWK	MXL	PUR	TSI	YRI
PKD1L3	rs147505098	9 (AIG) / 84 (GIG)	5 (AIG) / 84 (GIG)	1 (AIG) / 13 (GIG)	89 (GIG)	97 (GIG)	66 (GIG)	1 (AIG) / 54 (GIG)	3 (AIG) / 95 (GIG)	88 (GIG)
	rs4788587	8 (AIA) / 30 (AIG) / 55 (GIG)	2 (AIA) / 24 (AIG) / 63 (GIG)	5 (AIG) / 9 (GIG)	10 (AIA) / 41 (AIG) / 38 (GIG)	5 (AIA) / 34 (AIG) / 58 (GIG)	4 (AIA) / 18 (AIG) / 44 (GIG)	4 (AIA) / 16 (AIG) / 35 (GIG)	4 (AIA) / 25 (AIG) / 69 (GIG)	4 (AIA) / 37 (AIG) / 47 (GIG)
PKD2L1	rs7904983	93 (GIG)	89 (GIG)	14 (GIG)	89 (GIG)	6 (AIA) / 41 (AIG) / 50 (GIG)	3 (AIG) / 63 (GIG)	4 (AIG) / 51 (GIG)	1 (AIG) / 97 (GIG)	4 (AIA) / 27 (AIG) / 57 (GIG)
TAS1R1	rs149094445	93 (CIC)	89 (CIC)	14 (CIC)	89 (CIC)	97 (CIC)	66 (CIC)	55 (CIC)	98 (CIC)	88 (CIC)
	rs140193828	93 (GIG)	1 (AIG) / 88 (GIG)	14 (GIG)	89 (GIG)	97 (GIG)	66 (GIG)	55 (GIG)	98 (GIG)	88 (GIG)
	rs115030111	93 (GIG)	89 (GIG)	14 (GIG)	89 (GIG)	97 (GIG)	66 (GIG)	55 (GIG)	98 (GIG)	1 (AIG) / 87 (GIG)
TAS1R3	rs141631187									
TAS2R3	rs138466830	93 (GIG)	89 (GIG)	14 (GIG)	89 (GIG)	97 (GIG)	66 (GIG)	55 (GIG)	98 (GIG)	88 (GIG)
TAS2R7	rs150192473	93 (GIG)	89 (GIG)	14 (GIG)	89 (GIG)	97 (GIG)	8 (AIG) / 58 (GIG)	1 (AIG) / 54 (GIG)	98 (GIG)	88 (GIG)
TAS2R8	rs41324347	93 (CIC)	89 (CIC)	14 (CIC)	89 (CIC)	1 (AIA) / 2 (AIC) / 94 (CIC)	1 (AIC) / 65 (CIC)	55 (CIC)	98 (CIC)	7 (AIC) / 81 (CIC)
TAS2R14	rs34789740	93 (GIG)	89 (GIG)	14 (GIG)	89 (GIG)	97 (GIG)	66 (GIG)	55 (GIG)	98 (GIG)	1 (AIG) / 87 (GIG)
TAS2R19	rs77837442	91 (CIC) / 2 (CIT)	86 (CIC) / 3 (CIT)	13 (CIC) / 1 (CIT)	89 (CIC)	97 (CIC)	62 (CIC) / 4 (CIT)	54 (CIC) / 1 (CIT)	97 (CIC) / 1 (CIT)	88 (CIC)
TAS2R20	rs116400924	93 (CIC)	89 (CIC)	14 (CIC)	89 (CIC)	97 (CIC)	66 (CIC)	55 (CIC)	98 (CIC)	83 (CIC) / 5 (CIT)
TAS2R40	rs145138257	93 (CIC)	89 (CIC)	14 (CIC)	89 (CIC)	97 (CIC)	66 (CIC)	55 (CIC)	96 (CIC) / 2 (CIT)	88 (CIC)
TAS2R41	rs201235995									
TAS2R42	rs141369006	93 (CIC)	89 (CIC)	14 (CIC)	89 (CIC)	96 (CIC) / 1 (CIT)	66 (CIC)	54 (CIC) / 1 (CIT)	98 (CIC)	88 (CIC)
TAS2R43	rs113710768									
TAS2R46	rs150894148									
	rs2708381	54 (CIC) / 31 (CIT) / 8 (TIT)	55 (CIC) / 31 (CIT) / 3 (TIT)	9 (CIC) / 5 (CIT)	58 (CIC) / 27 (CIT) / 4 (TIT)	67 (CIC) / 26 (CIT) / 4 (TIT)	36 (CIC) / 30 (CIT)	34 (CIC) / 18 (CIT) / 3 (TIT)	65 (CIC) / 30 (CIT) / 3 (TIT)	51 (CIC) / 35 (CIT) / 2 (TIT)
TAS2R60	rs138984388	93 (CIC)	89 (CIC)	14 (CIC)	89 (CIC)	97 (CIC)	66 (CIC)	55 (CIC)	98 (CIC)	88 (CIC)
SCNN1D	rs148588796	93 (CIC)	89 (CIC)	14 (CIC)	89 (CIC)	96 (CIC) / 1 (CIT)	66 (CIC)	55 (CIC)	98 (CIC)	88 (CIC)
CD36	rs114881528	93 (GIG)	89 (GIG)	14 (GIG)	89 (GIG)	97 (GIG)	66 (GIG)	55 (GIG)	98 (GIG)	1 (AIG) / 87 (GIG)
	rs3211893	93 (TIT)	89 (TIT)	14 (TIT)	89 (TIT)	3 (CIT) / 94 (TIT)	66 (TIT)	55 (TIT)	98 (TIT)	4 (CIT) / 84 (TIT)
	rs3211938	93 (TIT)	89 (TIT)	14 (TIT)	89 (TIT)	12 (GIT) / 85 (TIT)	66 (TIT)	55 (TIT)	98 (TIT)	8 (GIG) / 35 (GIT) / 45 (TIT)
	rs56381858									

Table S4

Comparison of single nucleotide mutation of taste receptors between modern and archaic humans (Neanderthal and Denisovan) and their estimated allele age. Normal and bold font in modern humans section indicate the mutant and normal allele, respectively.

Table S4

							Constant population		Population growth	
**Gene**	**NCBI.37**	**rsID**	**Mutation type**	**Modern human**	**Neanderthal**	**Denisovan**	**EA Est. Age (kyrs)**	**AA Est. Age (kyrs)**	**EA Est. Age (kyrs)**	**AA Est. Age (kyrs)**
PKD1L3	NM_181536.1	rs147505098	stop-gained	A/**G**	G	G	7.1 ± 23.8	0.8 ± 2.3	NA	NA
		rs4788587	stop-gained	A/**G**	G	G	31.9 ± 65.4	14.8 ± 32.9	NA	NA
PKD2L1	NM_016112.2	rs7904983	stop-gained	A/**G**	G	G	1.7 ± 4.3	39.5 ± 79.1	7.1 ± 27.0	301.0 ± 286.6
TAS1R1	NM_138697.3	rs149094445	stop-gained	T/**C**	C	C	NA	3.3 ± 8.8	NA	NA
		rs140193828	stop-gained	A/**G**	G	G	1.1 ± 2.9	NA	NA	NA
		rs115030111	stop-gained	A/**G**	G	G	0.2 ± 0.6	2.1 ± 6.3	1.2 ± 3.3	22.3 ± 47.3
TAS1R3	NM_152228.1	rs141631187	5'splice	A/**G**	G	G	NA	0.4 ± 1.1	NA	4.2 ± 10.8
TAS2R3	NM_016943.2	rs138466830	stop-gained	A/**G**	G	G	2.9 ± 8.0	0.7 ± 2.0	14.6 ± 50.1	8.1 ± 20.4
TAS2R7	NM_023919.2	rs150192473	stop-gained	A/**G**	G	G	0.2 ± 0.6	0.4 ± 1.1	1.2 ± 3.3	4.2 ± 10.8
TAS2R8	NM_023918.1	rs41324347	stop-gained	A/**C**	C	C	0.2 ± 0.6	11.1 ± 30.2	1.2 ± 3.3	89.2 ± 127.9
TAS2R14	NM_023922.1	rs34789740	stop-gained	A/**G**	G	G	NA	2.5 ± 6.8	NA	24.3 ± 50.5
TAS2R19	NM_176888.1	rs77837442	stop-gained	T/**C**	C	C	12.4 ± 33.0	1.9 ± 5.2	60.6 ± 122.4	20.2 ± 43.8
TAS2R20	NM_176889.2	rs116400924	stop-gained	T/**C**	C	C	0.2 ± 0.6	5.6 ± 15.5	1.2 ± 3.3	48.9 ± 83.8
TAS2R40	NM_176882.1	rs145138257	stop-gained	T/**C**	C	C	1.4 ± 4.0	NA	NA	NA
TAS2R41	NM_176883.2	rs201235995	stop-gained	A/**G**	G	G	NA	1.1 ± 3.1	NA	NA
TAS2R42	NM_181429.1	rs141369006	stop-gained	T/**C**	C	C	NA	1.6 ± 4.3	NA	19.1 ± 41.9
TAS2R43	NM_176884.2	rs113710768	stop-gained	T/**C**	C	C	79.2± 120.1	25.1 ± 54.1	NA	NA
TAS2R46	NM_176887.2	rs150894148	stop-gained	A/**G**	G	G	NA	4.5 ± 12.2	NA	NA
		rs2708381	stop-gained	T/**C**	C	C	93.6 ± 150.1	48.2 ± 83.4	341.7 ± 324.1	347.7 ± 309.6
TAS2R60	NM_177437.1	rs138984388	stop-gained	G/**C**	C	C	0.2 ± 0.6	1.4 ± 4.0	1.2 ± 3.3	16.8 ± 37.8
SCNN1D	NM_001130413.3	rs148588796	stop-gained	T/**C**	C	C	NA	1.1 ± 3.1	NA	12.9 ± 30.5
CD36	NM_000072.3	rs114881528	5'splice	A/**G**	G	G	NA	0.5 ± 1.3	NA	6.2 ± 15.9
		rs3211893	5'splice	C/**T**	T	T	NA	2.9 ± 8.0	NA	28.1 ± 56.4
		rs3211938	stop-gained	G/**T**	T	T	0.4 ± 1.1	24.6 ± 50.5	1.2 ± 3.3	182.6 ± 210.3
		rs56381858	stop-gained	G/**T**	T	T	0.4 ± 1.1	NA	4.3 ± 15.6	NA
